# Re: Sim et al: Additive Effect of Highly Aspherical Lenslet Target Spectacles to Children Inadequately Controlled by Atropine

**DOI:** 10.1016/j.xops.2025.100982

**Published:** 2025-10-22

**Authors:** Hakan Kaymak, Berthold Seitz, Michael Bärtschi, Machteld Devenijn

**Affiliations:** 1 Gottfried O.H. Naumann Professor for Epidemiology and Prevention of Myopia, Saarland University, Homburg, Germany; 2 Department of Ophthalmology, Saarland University Hospital, Homburg, Germany; 3 Eyeness AG, Bern, Switzerland; 4 Internationale Innovative Ophthalmochirurgie GbR, Duesseldorf, Germany

TO THE EDITOR:

With great interest, we read the article by Sim et al, who investigated the effects of combining highly aspherical lenslet target (HALT) spectacle lenses with low-dose atropine (LDA) in children whose myopia progressed despite pharmacological monotherapy. The study demonstrates a significant reduction in both axial elongation and refractive progression following the addition of HALT lenses. However, we wish to raise a conceptual concern regarding the interpretation of these findings.

**Figure 1:**
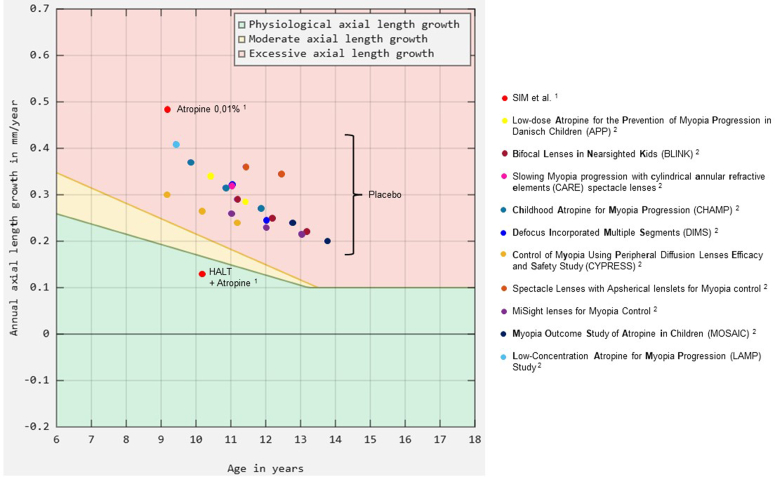
Annual axial length growth and therapeutic classification zones according to Graff et al. HALT = highly aspherical lenslet target.

The entire cohort consisted of children who had already shown inadequate response to LDA (≥0.5 D progression within 6 months). The axial growth during LDA monotherapy averaged 0.24 mm in 6 months (≈0,48 mm/year)^1^—well above the range observed in placebo arms of previous trials,^2^ illustrated by Figure 1. Figure 1 shows the mean annual axial elongation of the control arms from published randomized myopia trials adapted from Brennan et al, as well as the results from Sim et al. The background color zones reflect normative growth boundaries adapted from Graff et al.^3^ In this context, it is difficult to assert a synergistic benefit of the combination, as 0.01% atropine likely contributed very little to the outcome.

Following the addition of HALT lenses, axial elongation dropped to 0.06 mm at 6 months and 0.14 mm at 12 months. While this reduction is noteworthy, the absence of a HALT-only control group makes it impossible to determine whether this improvement is attributable to a true pharmacologic–optical synergy or merely to the effect of HALT alone. Notably, the pharmacological component—0.01% atropine—did not even produce a measurable effect on pupil size in this cohort, unlike in previous studies, raising further doubts about its contribution. More fundamentally, no combination therapy study should claim superiority without the inclusion of a contemporaneous placebo group. Historical comparisons—even in prospective designs—introduce bias due to natural age-related deceleration of eye growth.^4^

Moreover, the continued use of refractive error (spherical equivalent) as a primary outcome remains problematic. Numerous studies have shown that refractive change correlates only loosely with axial elongation, especially in younger children.^5^ If the goal of therapy is to reduce structural risk and prevent myopia-associated complications, then axial length and the achievement of emmetropic growth should serve as the benchmark.^3^

The study’s strength lies in its practical applicability and its focus on LDA-refractory eyes. Still, it may unintentionally call into question the utility of 0.01% atropine as a first-line intervention when potent optical strategies like HALT are available.

We therefore propose that future studies:

1. include a HALT monotherapy arm,
2. define physiological emmetropic growth as the therapeutic target, and
3. always incorporate a contemporaneous placebo group.

Such refinements are essential for a meaningful and ethically robust evaluation of (combination) therapies in pediatric myopia.^3^

Furthermore, given that all children included in the study had already shown insufficient response to atropine monotherapy, it is worth reflecting on the rationale for continuing a pharmacological treatment that had likely failed. If 0.01% atropine contributed little to controlling axial elongation during the first 6 months, one must ask whether it is ethically sound to maintain such a treatment without clear evidence of benefit. Continuing ineffective therapy may delay access to more effective interventions and potentially mislead families regarding its expected efficacy. As long as no therapeutic target is defined, success cannot be meaningfully classified—even in real-life studies. Clinical inertia, especially when unsupported by mechanistic plausibility or prior success, may lead to false conclusions regarding the effectiveness of a therapy and thereby distort both clinical decision-making and scientific interpretation.

Sincerely
